# International Standards for the 12-Minute Cooper Test on a Concept 2 Rowing Ergometer: Validity and Reliability of the Test

**DOI:** 10.5114/jhk/195638

**Published:** 2025-05-29

**Authors:** Robert Podstawski, Krzysztof Borysławski, Ferenc Ihász, Piotr Gronek

**Affiliations:** 1 Department of Physiotherapy, Human Wellness Research Laboratory, University of Warmia and Mazury in Olsztyn, Olsztyn, Poland.; 2 Institute of Health, Angelus Silesius University of Applied Sciences, Wałbrzych, Poland.; 3 Faculty of Psychology and Pedagogy, Institute of Sports Sciences, Eötvös Lóránd University, Szombathely, Hungary.; 4 Department of Dance, University of Physical Education in Poznań, Poznań, Poland.

**Keywords:** motor abilities, motor test, fitness, norms, university students, testing

## Abstract

The aim of this study was to develop international standards for evaluating strength endurance abilities with the use of the 12-min Cooper Test on a rowing ergometer (12-MCTRE). The results from 1855 men (1421 from Poland and 434 from Hungary) and 2879 women (2502 from Poland and 412 from Hungary) aged 19–25 (mean age of 19.98 ± 1.04 and 20.36 ± 0.94, respectively) were collated between 2004 and 2020. The results were expressed on a uniform T-score scale for the 12-MCTRE. The validity of the 12-MCTRE was verified on a group of 28 male and 32 female participants based on the registered variables (power [W], energy expenditure [Kcal], training intensity distribution, and sexual dimorphism). On average, men covered a distance of 2535.6 m and women covered a distance of 1581.0 m during the 12-MCTRE. The results of the control group indicate that men covered a significantly (p < 0.001) longer distance (2691.5 m) than women (2116.1 m) in the 12-MCTRE, and the values of the remaining variables (power, energy expenditure, pace/500 m, and HR_max_) were also significantly better in male participants. During the 12-MCTRE, both women and men remained in the very high effort zone for the longest period of time (516.9 s and 363.1 s, respectively). Women spent significantly more time (p < 0.001) in the high effort zone than men, but significantly less time in the maximal effort zone and the very high effort zone. The 12-MCTRE is an effective tool for evaluating strength endurance abilities in young adults.

## Introduction

It is well known that physical activity constitutes one of the key determinants of a healthy lifestyle, and it delivers benefits not only for physical health, but also for social and mental health (Norton et al., 2010; Olszewska et al., 2017). Prospective observational studies have demonstrated that regular physical activity is inversely correlated with disease outcomes, including cardiovascular disease, thromboembolic stroke, hypertension, type 2 diabetes mellitus, osteoporosis, obesity, colon cancer, breast cancer, anxiety, and depression (Haskel et al., 2007). Compelling new evidence has also been provided by studies of women (Greeg et al., 2003), stroke patients (Gallanagh et al., 2011), as well as patients affected by some types of cancers (McTiernan et al., 2019) and cognitive impairment (Mandolesi et al., 2018). Adequate and recommended levels of physical activity also affect motor abilities (Garber et al., 2011). Each component of motor fitness, including cardiorespiratory fitness, muscular strength and endurance, body composition, flexibility, and neuromotor fitness, has a beneficial impact on some aspects of health (American College of Sports Medicine, 1998).

Strength endurance abilities play a particularly important role in the group of motor abilities that affect physical health (Podstawski et al., 2019). Higher levels of cardiorespiratory and muscular fitness decrease health risks (Barlow et al., 2006; Cherouveim et al., 2022; Church et al., 2005; Fitz Gerald et al., 2004). Cardiorespiratory exercise reduces several risks associated with cardiometabolic diseases, although the magnitude of this effect is modest and tends to depend on individual characteristics and the exercise program, where a change in one cardiometabolic risk factor occurs independently of changes in other factors (Thompson et al., 2003). In middle-aged individuals and seniors whose physical activity levels meet qualitative and quantitative recommendations, improvements have been observed in hypertension, glucose intolerance, insulin resistance, dyslipidemia, and inflammatory markers, also during regaining of weight (Booth et al., 2000; Weis and Holloszy, 2007). In turn, improvements in muscular strength have been shown to significantly reduce cardiometabolic risks (Jurca et al., 2004), the risk of all-cause mortality (Fitz Gerald et al., 2004), cardiovascular risks (Gale et al., 2007), and the risk of functional limitations (Bårdstu et al., 2022) and nonfatal diseases (Clael and Bezerra, 2019).

Assessments of motor abilities help monitor and promote physical activity and improve the efficacy of the implemented exercise programs (Uysal et al., 2023). Physical activity programs should follow specific guidelines to minimize potential risks, including the intensity of exercise that exceeds the participants' physical ability or high relative intensity of exercise for persons with low fitness levels (Norton et al., 2010). For this reason, motor fitness tests are a valuable tool for assessing physical health. In adults, motor fitness tests should be conducted regularly (for example, every six months), and they should be accompanied by body composition analyses and medical exams (Kherada and Carneirob, 2023). Therefore, tests evaluating motor abilities (as components of the overall motor fitness) play an important role in health programs and should be conducted with the use of specifically designed motor tests (Podstawski and Żurek, 2022). The aim of this study was to propose international motor fitness standards and to evaluate strength endurance abilities in university students. In addition, the participants' physiological characteristics and sexual dimorphism were analyzed in detail during the study to determine the validity of the applied test.

## Methods

### Participants

The presented classification standards were developed in a study that was conducted in 2004–2020 on a large, representative, and random population sample. Potential participants were informed about the purpose of the study during obligatory physical education (PE), physiology and anthropomotorics classes. The results from 1855 men (1421 from Poland and 434 from Hungary) and 2879 women (2502 from Poland and 412 from Hungary), aged 19–25 (mean age of 19.98 ± 1.04 and 20.36 ± 0.94, respectively) were collated between 2004 and 2020. The study was conducted at the University of Warmia and Mazury in Olsztyn, Poland, and the Eötvös Lóránd University in Szombathely, Hungary. International classification standards were applied to ensure the objectivity of the study. Inclusion and exclusion criteria were selected using the methodology proposed by Podstawski et al. (2015). The study involved students attending obligatory PE classes of 90 min per week. Students participating in extracurricular physical activities (members of sports teams) were not included in the study because their performance could significantly affect the results of this study. Students who were absent on the day of the evaluation or were not willing to participate were not included in the study either. A total of 157 women and 123 men presented medical certificates or had permanent damage to locomotor organs, and 100 women and 57 men refused to participate without providing a reason and therefore did not take part in the test.

### Procedures

The research was performed in compliance with the Declaration of Helsinki and upon the prior consent of the Ethics Committee of the University of Warmia and Mazury in Olsztyn (decision No. 39/2011; approval date: 24 November 2011). All participants gave their written consent to participate in the study.

### Measurements

Body height measurements were performed to the nearest 0.1 mm on a WB-150 medical scale with a stadiometer and a Martin anthropometer based on standard guidelines. Body mass was determined to the nearest 0.1 kg, and the results were used to calculate the participants' BMI. Strength endurance abilities were evaluated with the use of a 12-min test on a Concept 2 rowing ergometer (12-MCTRE) (Podstawski et al., 2022). During preliminary meetings held before the study, each participant performed one 12-MCTRE per week. Five trials of the 12-MCTRE were conducted to guarantee reliable results. Participants were provided with links to online instructional videos and rowing exercise programs.

Participants were instructed on how to correctly perform the 12-MCTRE before the commencement of the study, and they were allowed time to practice. The test was preceded by an active warm-up (10 min). The warm-up routine included jogging, along with general and specific resistance and stretching exercises (Frandkin et al., 2010).

### Correct Technique for Performing the 12-Minute Cooper Test on a Rowing Ergometer

**Figure 1:**
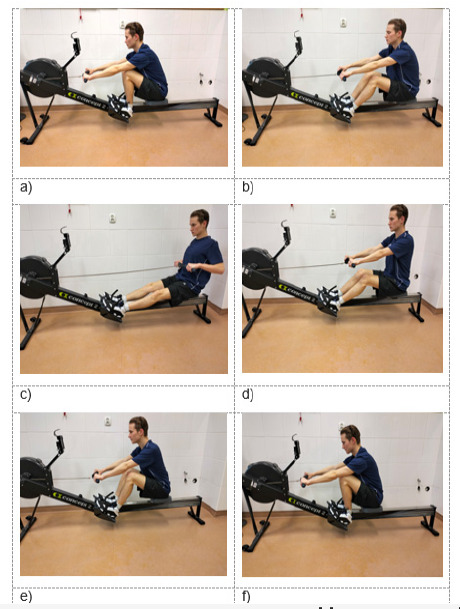
Rowing phases on a Concept 2 PM rowing ergometer.

The participants' strength endurance abilities were evaluated based on the distance (in meters) covered during the 12-MCTRE. The row stroke was divided into two phases according to the technique presented by Podstawski et al. (2022) (Figure 1).

**Phase I (pulling the bar):** from a starting position with the legs bent, arms straight, and the back leaning forward (Figure 1a), the lower limbs were gradually straightened (Figure 1b), the torso was abducted backward to a maximum of 45° (Figure 1c), and the bar was pulled to the chest (Figure 1d).

**Phase II (bar inversion):** from a position with the legs straight and the torso abducted and pulled back (Figure 1d), the arms were extended, the torso was bent forward (Figure 1c), and the lower limbs were flexed to a position with maximally bent legs, with the torso bent forward and the arms straight (Figure 1a).

When pulling the bar, the movement began with the lower limbs extended and the torso abducted backward, and it ended with the arms flexed and the bar pulled to the chest (between the nipples and the xiphoid process). During bar inversion, the movement began with the arms straight and the torso bent forward, and it ended with the knees bent (and the heels as close as possible to the buttocks).

Participants completed the distance within the given time limit (12 min).

The results of the control group (28 males and 32 females) during the 12-MCTRE performed on a Concept 2 PM5 standardized rowing ergometer (PH Markus, Szczecin, Poland), which is widely used to measure strength endurance abilities in athletes (Alfőldi et al., 2021), were analyzed. The following variables were measured during the rowing ergometer test: maximum, average, and minimum heart rate (HR_max, avg, min_), total distance covered within 12 min, total power generated during the test [W], average time to 500 m, the calorie burn rate [kcal] per hour, strokes per minute (SPM) [s/m]. Each participant wore a Polar H10 heart rate sensor (Polar Electro Oy, Kempele, Finland) on a chest strap. The ergometer was programmed and paired with the ErgData application and the HR sensor. The test was initiated upon a verbal start cue. The system was automatically activated when the handle was engaged and automatically deactivated after 12 min. The data were logged into the app. The data from the ErgData app were recorded in an Excel spreadsheet.

### Statistical Analysis

Minimum, maximum, and average values and standard deviations were determined in a statistical analysis. Percentile ranks were calculated for the results reached by every participant. The standards for evaluating strength endurance abilities were expressed as T-score values and presented on a five-point grading scale (very poor, poor, average, good, very good). Sexual dimorphism in the control sample was analyzed using the Student's *t*-test for independent samples (the Shapiro-Wilk test revealed that all analyzed variables followed a normal distribution). The obtained results were used to classify the participants' fitness levels. These data can be also used to monitor changes in the strength endurance abilities of a population.

## Results

On average, men covered a distance of 2535.6 m and women covered a distance of 1581.0m during the 12-MCTRE (Table 1). The BMI of both female and male participants was within the norm (23.3 and 22.1 kg/m^2^, respectively). The results reached by men and women are presented on a five-point grading scale (with a tolerance range for each score) in Table 2.

**Table 1:** Descriptive statistics of the studied population.

Characteristics	Males (N = 1855)			Females (N = 2879)		
	Mean	SD	min-max	Mean	SD	min-max
**Age [years]**	19.98	1.04	19–25	19.58	0.75	19–24
**Body height [cm]**	181.12	6.08	160.2–202.2	165.06	6.47	139.8–195.0
**Body mass [kg]**	76.57	9.94	55.2–120.6	59.11	7.79	40.0–101.3
**BMI [kg/m^2^]**	23.34	2.79	16.22–6.33	21.76	3.16	15.02–38.22
**Distance covered [m]**	2535.57	311.01	1445–2999	1580.96	230.03	489–2166

**Table 2:** Five-point grading scale for evaluating female and male participants.

Level	Tolerance range		N
	Meters	Points	
**Males**			
**Very poor**	1445–2224	1÷20	371
**Poor**	2225–2554	21÷40	371
**Average**	2555–2682	41÷60	379
**Good**	2683–2784	61÷80	376
**Very good**	2785–2999	81÷100	367
**Females**			
**Very poor**	489–1372	1÷20	577
**Poor**	1373–1603	21÷40	580
**Average**	1604–1684	41÷60	588
**Good**	1685–1761	61÷80	566
**Very good**	1762–2166	81÷100	568

results of the control group (Table 3) indicate that men covered a significantly (*p* < 0.001) longer distance (2691.5 m) than women (2116.1 m) during the 12-MCTRE. The values of the remaining variables, including power, energy expenditure, pace/500 m (lower values are better), and HR_max_ were also significantly (*p* < 0.05) better in men, excluding S/M and HR_avg_, where significant differences were not observed between the sexes (Table 3).

**Table 3:** Descriptive statistics for the 12-MCTRE.

Parameter	Males				Females				Difference	
	Mean	SD	Min.	Max.	Mean	SD	Min.	Max.	*t*	*p*
Distance [m]	2691.50	235.053	2060	3067	2116.13	182.05	1775	2557	10.67	<0.001
Power [Watts]	149.82	35.54	79	216	71.34	17.40	42	125	11.08	<0.001
Energy expenditure [Kcal]	161.00	24.98	105	207	107.59	12.75	88	146	10.62	<0.001
Strokes per minute (S/M)	28.54	3.67	20	38	26.88	3.50	20	33	1.79	ns
Pace/500m [s]	133.18	14.02	104	174	168.47	17.42	105	202	−8.56	<0.001
HR _avg_ [bpm]	175.04	12.65	139	197	169.66	12.88	121	193	1.62	ns
HR _max_ [bpm]	194.07	10.49	167	219	186.84	10.87	144	205	2.61	0.011

Training intensity distribution in male and female participants is presented in Table 4. During the 12-MCTRE, both women and men remained in the very high effort zone for the longest period of time (516.9 s and 363.1 s, respectively), compared with the remaining zones. Men spent least time in the very low effort zone (1.9 s), whereas women spent least time in the maximal effort zone (0.9 s). Women remained significantly longer (*p* < 0.001) in the high effort zone than men (99.7 s vs. 243.5 s, difference of 143.8 s), but significantly shorter in the maximal effort zone (difference of 9.2 s) and the very high-effort zone (difference of 153.8 s). In the remaining effort zones, the differences between the sexes were not significant.

**Table 4:** Training intensity distribution during the 12-MCTRE.

Intensity zones [bpm]		Males				Females				Difference		
		Mean	SD	Min.	Max.	Mean	SD	Min.	Max.	*t*	*p*
**Maximal: > 190**	s	10.1	17.2	0	62	0.9	2.2	0	12	2.97	0.004
	%	1.4	2.4	0	8.6	0.1	0.3	0	1.7		
**Very high: 173–190**	s	516.9	204.6	0	702	363.1	261.7	0	697	2.51	0.015
	%	71.8	28.4	0	97.5	50.4	36.3	0	96.8		
**High: 153–172**	s	99.7	106.1	5	423	243.5	211.1	0	603	−3.26	0.002
	%	13.9	14.7	1	58.7	33.8	29.3	0	83.8		
**Moderate: 134–152**	s	65.6	120.2	0	514	71.6	83.8	0	355	−0.23	ns
	%	9.1	16.7	0	71.4	9.9	11.6	0	49.3		
**Low: 115–133**	s	25.6	84.2	0	449	29.3	78.1	0	403	−0.18	ns
	%	3.6	11.7	0	62.4	4.1	10.8	0	55.9		
**Very low: 104–114**	s	1.9	7.7	0	40	11.6	53.7	0	303	−0.94	ns
	%	0.3	1.1	0	5.6	1.6	7.5	0	42.1		

## Discussion

The 12-MCTRE administered in the present study was modeled on the Cooper's 12-min run test (CRT). Research has shown that the original CRT is a valid and reliable motor test for measuring aerobic (cardiorespiratory) fitness (Bandyopadhyay, 2015; Penry et al., 2011). In turn, rowing has been described as a cyclic sport that typically consists of both strength and endurance sessions (Alfoldi et al., 2021). Hybrid exercise training is characteristic of rowing regattas over a standardized distance of 2 km, where each race lasts 5.5 to 7 min (Mikulić, 2008). In rowing competitions, relative anaerobic contribution ranges from 21 to 30% (Secher, 1993), whereas the aerobic metabolism contributes 75–80% of the energy demand (Juriśić et al., 2014). In the 12-MCTRE, the duration of effort is approximately twice longer, which indicates that endurance abilities play a more important role than strength abilities relative to a 2-km rowing distance in regattas. Therefore, the endurance component in strength endurance abilities is dominant in the 12-MCTRE. The present study fills in the knowledge gap concerning the Cooper test, and it indicates that the 12-MCTRE is a useful tool for assessing strength endurance abilities in young adults.

The 12-MCTRE can be successfully implemented in functional training programs, in particular in aerobic training. The health benefits associated with aerobic and strength training were described in the Introduction. This section focuses on improvements in health that are directly associated with training on a Concept 2 rowing ergometer. The ergometer features a sliding seat which reduces the load on the lower limbs during exercise. In the Concept 2 rowing ergometer, the load can be additionally controlled by adjusting resistance on a 10-point scale. This option can be used by persons with excessive weight or persons with decreased functional mobility of the lower limbs to make rowing more of a strength or an endurance workout. Research has demonstrated that around 75–80% of the power produced by successful elite rowers during sliding-seat rowing is generated by the lower limbs, whereas 20–25% is generated by the torso and the arms (Cosgrove et al., 1999). Therefore, the grading scale proposed in this study can be used to assess endurance and strength abilities in both healthy subjects and persons who cannot be submitted to run tests.

An assessment of sexual dimorphism in the 12-MCTRE revealed that men significantly (*p* < 0.001) outperformed women in terms of physiological characteristics (distance covered, power, energy expenditure). Previous research has shown that men perform better than women in rowing competitions (Keenan et al., 2018; Podstawski et al., 2022), although sexual dimorphism in rowing has been rarely examined in the literature. The present study also confirmed that men achieved higher maximal and submaximal effort levels than women (Hunter and Stevens, 2013; Tanaka and Seals, 2008).

Due to the discussed advantages of a rowing ergometer, further research is needed to determine the extent to which this equipment can be used in functional training to improve motor abilities of professional athletes in various sports disciplines, as well as individuals who exercise to derive health benefits. Limb injuries sustained by athletes and sedentary individuals often require physiotherapy in positions that stabilize the affected joints. A rowing ergometer is an excellent piece of equipment that can be used in physiotherapy to stabilize knee, ankle, and hip joints.

In line with standard training practice, resistance training should be performed on different days than aerobic training, usually on two or three non-consecutive days of the week. According to experts, resistance training exercises targeting the major muscle groups should be repeated 8–12 times with a one-repetition maximum (1RM) of 40–80%, depending on the person's training level. To enable recovery, a rest interval of 2–3 min is recommended between the exercises (Garber et al., 2011). Based on the standard guidelines for aerobic training, 150 min of moderate-intensity exercise (46–63% of maximal oxygen uptake, VO_2max_) (30–60 min per session) and/or 75 minutes of vigorous-intensity exercise (64–90% of VO_2max_) (20–60 min per session) are recommended per week (Garber et al., 2011). According to Klika and Jordan (2013), traditional protocols may be effective, but they are not realistic in adults. The above applies also to young adults, including university students who lack time for exercise (Carballo-Fazanes et al., 2020). For an average person, these protocols may be unattractive and difficult to implement because the standard recommendations for strength and endurance training require considerable time and involve certain logistic constraints. Therefore, from the point of view of health promotion, holistic training, including workouts that promote strength endurance abilities, are more likely to attract the interest of young adults and can be used to evaluate and improve their motor fitness and increase their physical activity levels.

## Conclusions

The 12-MCTRE is an effective tool for evaluating strength endurance abilities in young adults. Testing standards should be regularly revised and updated to guarantee and maintain maximum reliability. The results of this study suggest that after the development of the classification standards for the 12-MCTRE, the test can be deployed to determine the strength endurance abilities of individuals from different countries.
